# Integrated correlation analysis of the thickness of buccal bone and gingiva of maxillary incisors

**DOI:** 10.1590/1678-7757-2024-0018

**Published:** 2024-06-14

**Authors:** Zhuohong GONG, Guangqi GAO, Mengru SHI, Xuejing GAN, Gengbin CAI, Hongcheng CHEN, Cuijun LI, Zhuofan CHEN, Danying CHEN, Zetao CHEN

**Affiliations:** 1 Sun Yat-sen University Guanghua School of Stomatology Hospital of Stomatology Guangzhou China Sun Yat-sen University , Guanghua School of Stomatology , Hospital of Stomatology , Guangdong Provincial Key Laboratory of Stomatology, Guangzhou , China .; 2 Sun Yat-sen University Guanghua School of Stomatology Hospital of Stomatology Guangzhou China Sun Yat-sen University , Guanghua School of Stomatology , Hospital of Stomatology , Department of Oral Implantology, Guangzhou , China .; 3 Sun Yat-sen University Guanghua School of Stomatology Hospital of Stomatology Guangzhou China Sun Yat-sen University , Guanghua School of Stomatology , Hospital of Stomatology , Zhujiang New Town Dental Clinic, Guangzhou , China .

**Keywords:** Canonical correlation analysis, Gingiva, Alveolar bone, Dental implantation

## Abstract

**Objective:**

This study aimed to validate the integrated correlation between the buccal bone and gingival thickness of the anterior maxilla, and to gain insight into the reference plane selection when measuring these two tissues before treatment with implants.

**Methodology:**

Cone beam computed tomography (CBCT) and model scans of 350 human subjects were registered in the coDiagnostiX software to obtain sagittal maxillary incisor sections. The buccal bone thickness was measured at the coronal (2, 4, and 6 mm apical to the cementoenamel junction [CEJ]) and apical (0, 2, and 4 mm coronal to the apex plane) regions. The buccal gingival thickness was measured at the supra-CEJ (0, 1mm coronal to the CEJ) and sub-CEJ regions (1, 2, 4, and 6 mm apical to the CEJ). Canonical correlation analysis was performed for intergroup correlation analysis and investigation of key parameters.

**Results:**

The mean thicknesses of the buccal bone and gingiva at different levels were 0.64~1.88 mm and 0.66~1.37 mm, respectively. There was a strong intergroup canonical correlation between the thickness of the buccal bone and that of the gingiva (r=0.837). The thickness of the buccal bone and gingiva at 2 mm apical to the CEJ are the most important indices with the highest canonical correlation coefficient and loadings. The most and least prevalent subgroups were the thin bone and thick gingiva group (accounting for 47.6%) and the thick bone and thick gingiva group (accounting for 8.6%).

**Conclusion:**

Within the limitations of this retrospective study, the thickness of the buccal bone is significantly correlated with that of the buccal gingiva, and the 2 mm region apical to the CEJ is a vital plane for quantifying the thickness of these two tissues

## Introduction

Adequate buccal bone and gingival thickness of the anterior maxilla plays an essential role in the long-term aesthetic outcomes of immediate implant placement in the anterior maxilla, while insufficient buccal bone and gingiva are associated with a high risk of aesthetic complications. ^1 , 2^ In clinical practice, gingival thickness is measured primarily by using a periodontal probe, ^3^ endodontic files, and ultrasonic devices. ^4^ Meanwhile, the CBCT is the most used modality for the quantitative analysis of bone morphology. ^5^

However, the methods used to measure the thickness of the buccal bone and gingiva are limited since these two counterparts show uneven morphologies. ^6^ To fit with their uneven distribution, various reference points have been proposed, including the cementoenamel junction (CEJ), alveolar crest, ^7^ bottom of the sulcus, ^8^ and gingival margin. ^9^ Nonetheless, these arbitrary measurement references can hinder the formulation of a measurement standard and the creation of a unified treatment plan. In addition, the thickness of the buccal bone and its overlying gingiva is evaluated independently but not integratively. Even though it seems that the two adjacent tissues are independent indices, they are closely related either anatomically or clinically. Both tissues develop from the ectomesenchyme and are connected by perforating fibers. ^10^ Moreover, different gingival phenotypes display different responses in bone resorption. ^11 , 12^ Random measurement protocols without integrated analysis are prone to improper case selection and even aesthetic complications after dental implant treatments. ^3 , 13 , 14^ Under these circumstances, the thickness of the buccal bone and gingiva should be measured at the ideal reference plane in an integrated manner.

The standardized and unified analysis of the buccal bone and gingiva still encounters multiple challenges. The first roadblock is that the number of included patients is too small to support robust conclusions, as some measurement methods are invasive (e.g., the endodontic reamer) and difficult to repeat in clinical practice. Due to the diverse sources, researchers have failed to perform repeatable measurements of the buccal bone and gingiva at identical reference planes, and there are there are no widely acknowledged reference widely acknowledged reference planes. ^9^ Moreover, most studies have focused on the one-by-one analysis of two independent indices (i.e., one index from the buccal bone correlates with one from the gingiva), and few studies have analyzed the buccal bone and gingiva as two whole groups. ^14^ The deficiency of multivariable analysis has led to a lack of insight into which indices play important roles in the representation of the buccal bone and gingiva.

With the development of digital dentistry, digital methods (i.e., intraoral scans and model scans) that replicate real-world data show the potential to accelerate the data collection process and enlarge patient datasets. While the information from a single modality hinders the depiction of two tissues with different densities, it is necessary to superimpose the bone information from CBCT and gingival information from scan data to the same coordinate system to obtain integrated measurements of the same size, location, and direction. ^15^ Furthermore, to conduct multivariable analysis, researchers can take advantage of canonical correlation analysis (CCA) to comprehensively analyze the correlation between two sets of variables. ^16^ For this, two linear equations are adopted to represent the corresponding variable sets. Different combinations of canonical variables for each index in the two linear equations are tested for numerous iterations until the largest correlation coefficient is obtained between the two equations. During this process, the contribution of each index to the whole variable set was examined to determine the vital contributing factors. CCA has been used to explore the correlation between clinical factors and the severity of chronic obstructive pulmonary disease, ^17^ as well as to determine the relationship between sleep quality and nutritional status. ^18^

To provide the best-fitting evidence for presurgical evaluation and treatment decision-making regarding immediate implant placement, it is crucial to clarify the measurement protocol on a scientific basis. Therefore, this study aimed to perform a population-based and paired quantitative measurement of the thickness of the buccal bone and gingiva via the registration of CBCT and plaster model scan data. Furthermore, an integrated correlation analysis between the thickness of the buccal bone and gingiva was conducted for a robust conclusion. By doing so, this study aimed to uncover the key parameters from the two tissues to provide a reference for optimized measurements.

## Methodology

This study was performed following the Helsinki Declaration and received ethical approval (No. KQEC-2020-29-04) from the Medical Ethics Committee of the Hospital of Stomatology of Sun Yat-sen Univeristy. Due to the retrospective nature of this study, the requirement for informed consent from participants was waived. In this study, CBCT and model scans from the patient dataset of the Department of Oral Implantology, Hospital of Stomatology of Sun Yat-sen Univeristy, dating from Oct 1 ^st^ , 2019, to Mar 22, 2022, were retrospectively obtained. The inclusion criteria were as follows: patients who (a) were older than 18 years, (b) had natural anterior maxillary teeth, and (c) had an intact plaster model with a buccal vestibular groove. The exclusion criteria included (a) missing or incomplete anterior maxillary central or lateral incisors; (b) deformities of the tooth and supporting structure of the anterior maxillary teeth, including severe alveolar defects, soft tissue defects, periodontitis, crowded dentition, impacted teeth, root resorption, root fracture, periapical periodontitis, and gingival recession; (c) CBCT images with motion artifacts and metal artifacts, including orthodontic appliances or restorations; and (d) defects or damage to the plaster model and/or with evident bubbles.

**Figure 1:**
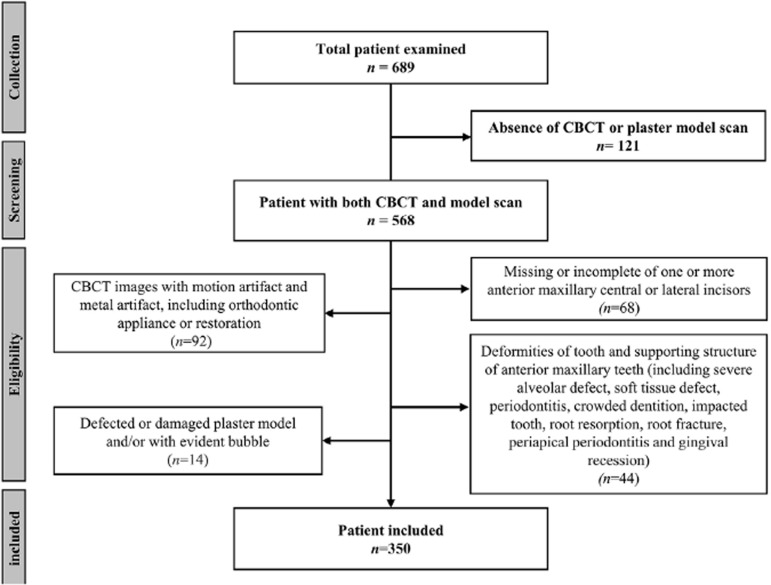
Flowchart of the patient inclution

CBCT images were acquired using a NewTom VG system (QR s.r.l.) with a voxel size of 0.3 mm and a 15 cm×12 cm field of view (FOV). The exposure parameters were automatically controlled by the machine. The Digital Imaging and Communications in Medicine (DICOM) files of the CBCT images were collected. The cast models were scanned three-dimensionally via a laboratory scanner (Ceramill Map 400; Amann Girrbach), and standard tessellation language (STL) files were produced ( Figure 1 ).

**Figure 2:**
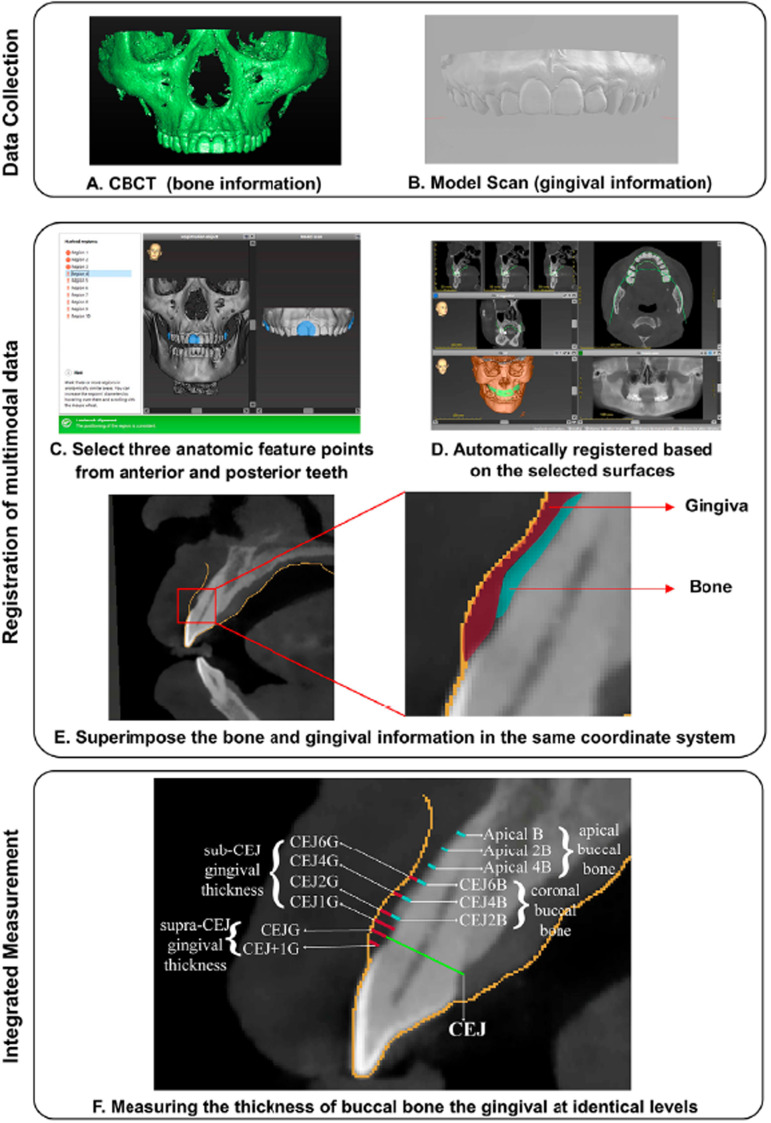
The schematics of the registration and integrated measurement method of the thickness of buccal bone and gingiva. The CBCT (containing bone information) and model scan (containing gingival information) data were first collected (A-B). The researcher imported both multimodal data into the coDiagnostiX software and selected three anatomic feature points from the anterior and posterior teeth (C). The software automatically registered the CBCT and model scan based on the selected surfaces (D). The sagittal planes of the incisors included the bone and gingival information in the same coordinate system (E). The measurement of buccal bone and gingiva was performed at preset identical levels (F). The measurement levels of buccal bone wall thickness were divided into the coronal buccal bone (2/4/6 mm apical to CEJ, abbreviated as CEJ2B, CEJ4B, and CEJ6B, respectively) and apical buccal bone (0/4/6 mm coronal to apex, abbreviated as ApicalB, Apical2B, and Apical4B, respectively). The measurement levels of buccal gingival thickness were divided into the supra-CEJ gingival thickness bone (at 1 mm apical to CEJ and at CEJ level, abbreviated as CEJ+1G and CEJG, respectively) and sub-CEJ gingival thickness (1/2/4/6 mm apical to CEJ, abbreviated as CEJ1G, CEJ2G, CEJ4G, and CEJ6G, respectively).

The DICOM and STL files from the same individual were imported into the implant planning software coDiagnostiX (Dental Wings). Then, one researcher manually selected three anatomic features (i.e., the mesial-incisal angle of the central incisors and the buccal surface of the left and right last molars), and coDiagnostiX software automatically registered these features based on the selected surfaces. ^15^ The registration result was three-dimensionally revised by a senior dentist (with more than 10 years of clinical experience). After, sagittal section images at the midfacial level of the maxillary central and lateral incisors were collected and saved in TIFF format ( Figure 2 ).

All sagittal images of the teeth were imported into Adobe Illustrator software (version 4.0, Adobe Systems Inc.). Index from sagittal images of all the teeth were measured by two well-trained researchers, with a high interrater coefficient (ICC), ranging from 0.581 to 0.821. Agreement was achieved by group meetings when there was inconsistency between the two researchers. The CEJ was set as the main reference plane. The measurements of buccal bone thickness included those of the coronal buccal bone group (2, 4, and 6 mm apical to the CEJ, abbreviated as CEJ2B, CEJ4B, and CEJ6B, respectively) and the apical buccal bone group (0, 2, and 4mm coronal to the apex plane, abbreviated as ApicalB, Apical2B, and Apical4B, respectively). The buccal gingival thickness was measured at the supra-CEJ (1 mm coronal to the CEJ and at the CEJ plane, abbreviated as CEJ+1G and CEJG, respectively) and sub-CEJ levels (1, 2, 4, and 6 mm apical to the CEJ, abbreviated as CEJ1G, CEJ2G, CEJ4G, CEJ6G, respectively) ( Figure 2 ).

The demographic characteristics of the included population were summarized. The statistical analysis of the data was performed using SPSS 26.0 (IBM Corp.). The quantitative data are presented as means and standard deviations (mean ± SD). Student’s t and Mann–Whitney tests were used to compare two independent samples with and without a normal distribution and variance equality. One-way ANOVA and Kruskal–Wallis test were used to compare three or more independent samples with and without normal distribution and variance equality. Canonical correlation analysis was performed for pairs of multivariate groups, and the canonical coefficients and loadings between each group were investigated. The chi-square test was used for correlation analysis between groups. The statistical significance level was set at α=0.05.

## Results

In this study, CBCT and cast model scan data from 689 patients were collected, and 350 patients (146 males and 204 females) were ultimately included according to the inclusion and exclusion criteria. The mean age of the included patients was 38.63±11.84 years, ranging from 20 to 78 years. A total of 1,400 anterior maxillary teeth were included, with 700 central and 700 lateral incisors.

**Figure 3:**
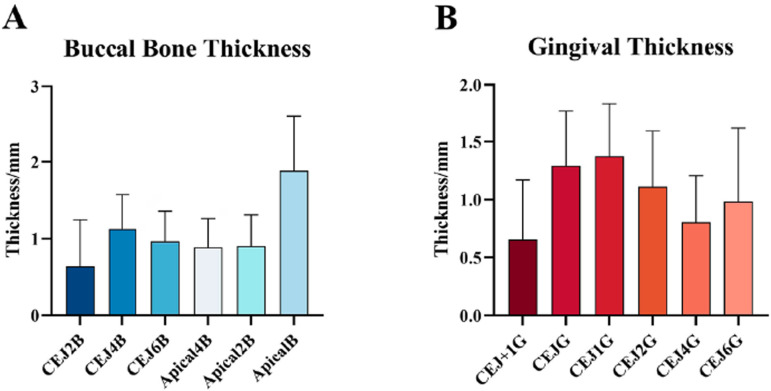
Overview of the distribution of buccal bone thickness and gingival thickness in the maxillary anterior region. The distribution of buccal bone thickness, with the thinnest thickness in the CEJ2B and two convexities in the CEJ4B and ApicalB (A). The distribution of gingival thickness, which peaks in the CEJ1G and concaves in CEJ4G (B).

The mean thickness of the buccal bone at different levels ranged from 0.64 mm to 1.88 mm. Notably, the value of CEJ2B was 0.64±0.61 mm, and 40% of the records were 0 mm at this level. The buccal bone wall at both the coronal level and the apical level was thicker than that at the mid-root level, except at 2 mm apical to the CEJ. The average buccal gingival thickness ranged from 0.66 mm to 1.37 mm. The thickest buccal gingiva was recorded 1 mm apical to the CEJ, namely, 1.37±0.46 mm ( Figure 3 ).

**Figure 4:**
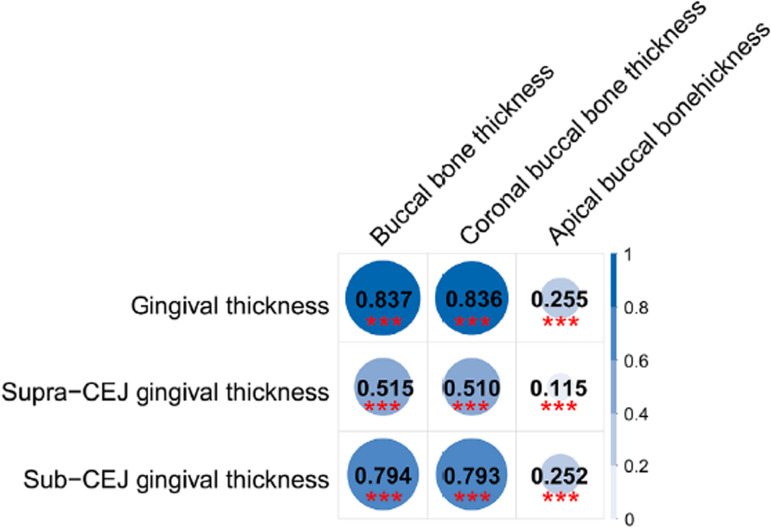
Heatmap of inter-group canonical correlation coefficient between the thickness of buccal bone and gingiva. The buccal bone thickness group (CEJ2B, CEJ4B, CEJ6B, Apical4B, Apical2B, ApicalB) is composed of the coronal buccal bone thickness (CEJ2B, CEJ4B, CEJ6B) and the apical buccal bone thickness (Apical4B, Apical2B, ApicalB). The gingival thickness group (CEJ+1G, CEJG, CEJ1G, CEJ2G, CEJ4G, CEJ6G) is composed of the supra-CEJ gingival thickness (CEJ+1G, CEJG) and sub-CEJ gingival thickness (CEJ1G, CEJ2G, CEJ4G, CEJ6G). *P<0.05, **P<0.01, ***P<0.001.

Canonical correlation analysis (CCA) revealed that buccal bone thickness was strongly correlated with buccal gingival thickness, with a canonical correlation coefficient of 0.837. Further, the subgroup CCA showed that the strong canonical correlation mainly lay between the coronal buccal bone thickness group ( *r* =0.836) and the sub-CEJ gingival thickness group ( *r* =0.794). On the other hand, there was also a strong correlation between the coronal buccal bone thickness group and the sub-CEJ gingival thickness group ( *r* =0.793) ( Figure 4 ). Furthermore, CEJ2B and CEJ2G presented the largest canonical correlation coefficient and canonical and cross loading in the CCA subgroup, with a high correlation ( *r* ≥0.60) (Table S1).

Young (18~29 y) females had the thickest CEJ2B, while old males had the thickest CEJ2G (Table S2). Considering 1 mm as the cutting point and the CEJ2B and CEJ2G as reference planes, the thick bone type composed 32.5% of the population of this study, and the thick gingival type composed 56.2%. Furthermore, the buccal soft and hard buccal tissue ensembles were subdivided into four subgroups: the thin gingiva and thin buccal bone (B-G-) subgroup (279 teeth, 19.9%), the thick gingiva and thin buccal bone (B-G+) subgroup (666 teeth, 47.6%), the thin gingiva and thick buccal bone (B+G-) subgroup (334 teeth, 23.9%), and the thick gingiva and thick buccal bone (B+G+) subgroup (121 teeth, 8.6%) ( Table 1 ). The mean age of the B-G+ subgroup (41.47±12.31 y) was the oldest, whereas that of the B+G- subgroup (35.25±10.24 y) was the youngest. Notably, the B+G+ subgroup contained more central incisors, and the B-G- subgroup contained more lateral incisors than did the other groups (Figure S1).

**Table 1:** Population distribution of thin and thick types of buccal bone and gingiva

Buccal bone and gingival thickness	Thick Buccal Bone(B+) (n, %)	Thin Buccal Bone(B-) (n, %)	Total teeth (n, %)
Thick Gingival Phenotype (G+) (n, %)	121(8.6%)	666(47.6%)	787(56.2%)
Thin Gingival Phenotype (G-) (n, %)	334(23.9%)	279(19.9%)	613(43.8%)
Total teeth (n, %)	455(32.5%)	945(67.5%)	1400(100%)

The Chi-square test showed a correlation between types of buccal bone thickness and buccal gingiva ( *P* <0.001)

## Discussion

In this study, the continuous uneven distributions of the buccal bone and gingival thicknesses were described based on a large population. This study validated the strong correlations between the thickness of the buccal bone and gingiva via canonical correlation analysis. Within the limitations of this retrospective study, the thicknesses of the buccal bone and gingiva 2 mm apical to the CEJ (i.e., CEJ2B and CEJ2G) were two key parameters of the corresponding tissues and may serve as references for measurement.

In this study, we performed a unified analysis of the buccal bone and gingiva via the registration of CBCT and model scans, and we propose a standardized measurement protocol for their thicknesses with a high intraclass correlation coefficient, which contributes to the large dataset of high quality. Compared to the relatively small dataset of related studies (in which the number of included teeth ranges from 10 to 598), ^14^ to the best of our knowledge, this study includes the largest number of patients (i.e., 350 patients with 1,400 incisors) based on a fully digital workflow. In addition, taking advantage of the registration of CBCT data and the cast model scans, this study fused the multimodal information into the same section, that is, the buccal bone from the CBCT and the soft tissue morphology from the model scan. Studies have shown that, when measuring gingival thickness, there is high interrater reliability agreement between the digital file superimposition method and the direct spring caliper method. ^19^ Therefore, the proposed method allows the integrated quantification of the buccal bone and gingiva at the same level with high reliability. Compared to the existing studies that performed separate measurements at different reference planes, the measurements from the same reference plane provided more details to prove the inherent correlation between the thickness of the buccal bone and gingiva.

This study revealed the uneven distribution of the buccal bone and gingiva and the complex dynamic changes in the coronal-apical plane. For the buccal bone, the thinnest part was recorded in the most coronal region (i.e., CEJ2B), followed by the convex region (i.e., CEJ4B). The buccal bone is relatively plain in the mid-root region (i.e., CEJ6B, Apical4B, and Apical2B); moreover, it is the thickest in the apical region. Similarly, researchers have also shown that the buccal bone at the mid-root level (0.89±0.34 mm) is thinner than that at the coronal (1.01±0.12 mm) and apical (1.4±0.52 mm) levels, while the fine-grained analysis in this study revealed a more complicated trend in the buccal bone thickness at different levels. ^6^ The thinnest gingiva is also recorded in the most coronal region (i.e., CEJ+1G), while the thickest lies on the CEJ1G. The gingiva concaves at 4 mm apical to the CEJ (i.e., the CEJ4G). The gingiva is thicker than 1 mm from the CEJ level to 2 mm apical to the CEJ.

To the best of our knowledge, this is the first study to validate the strong correlation between buccal bone thickness and gingival thickness via integrated analysis (i.e., CCA) and to further explore the key parameters of the two tissues (i.e., CEJ2B and CEJ2G). Some researchers have reported that a thick buccal bone is frequently observed in patients with a thick gingival phenotype. ^7^ A systematic review reported a positive correlation between the buccal bone thickness and the gingival thickness of the maxillary anterior teeth, the correlation coefficient of which ranged from 0.11 to 0.49. ^14^ In contrast, some studies have found a negative correlation ( *r* =−0.631~−0.691) between the thickness of the buccal bone and that of the gingiva in the esthetic zone. ^13^ Other studies have shown that the correlation exists only in specific regions, such as the right maxillary canine ^20^ or the maxillary second premolar. ^4^ In this study, the strong correlation between the two tissues was primarily shown in the CCA between the “buccal bone thickness” and “gingival thickness” groups, with the highest canonical correlation coefficient of 0.837. In the CCA subgroup, the coronal aspect of the buccal bone ( *r* =0.836) had a closer relationship with gingival thickness than did the apical aspect ( *r* =0.255). Similarly, the sub-CEJ gingival group ( *r* =0.794) had a closer relationship with the buccal bone than did the supra-CEJ group ( *r* =0.515). The coronal buccal bone thickness also exhibited a strong correlation with the sub-CEJ gingival thickness ( *r* =0.793). These CCA results narrow the key parameters to the coronal buccal bone and the subgingival region. A deeper investigation of the canonical and cross-loading between the bone-gingival CCA revealed that CEJ2B and CEJ2G presented the highest loading in each paired analysis. Thus, it can be concluded that the two key values (i.e., CEJ2B and CEJ2G) were the determining factors for the thickness of the buccal bone and gingiva, respectively.

It was intuitive that the most representative values were the thinnest buccal bone and gingiva, which were the CEJ2B and CEJ+1G in this study. However, the CEJ+1G showed weak relationships with other thicknesses of the buccal bone, leading to its lower representativeness than that of the CEJ2G. Considering that the CEJ is often located 1 mm submarginally, the CEJ-2 level refers to approximately 3 mm apical to the gingival margin. According to the 3A-2B rule, ^21^ the implant should be placed 3 mm from the cervical contour of the planned crown to achieve an appropriate biological width. Rojas-Vizcaya suggested that bone augmentation or reduction should be performed when the bone-margin distance is less than or greater than 3 mm. ^21^ Therefore, the soft and hard tissue at the CEJ2 level is clinically significant in the surgical planning and biological success of immediate implants and should be recognized as a standard reference for assessing the thickness of the buccal bone and gingiva in the esthetic zone.

It should be mentioned that these data present the baseline anatomic characteristics of periodontally healthy individuals, which may influence the clinical manifestations (i.e., probing depth, gingival recession, inflammation, attachment loss, etc.) of periodontal diseases, as well as the prognosis of prosthodontic treatments. ^22^ Based on the key parameters of CEJ2B and CJE2G, a total of four subclassifications of buccal bone and gingiva (B+G+, B-G-, B+G-, and B-G+) were performed to add a new dimension to comprehensively analyze the buccal osseous-gingival ensemble. The B+G+ and B-G- subgroups are in accordance with common sense, as they only comprised 8.6% and 19.9% of the included population, respectively. The B+G+ subgroup exhibits strong resistance to gingival recession, inflammation, and bone resorption ^23^ and is therefore favorable for immediate implant placement; ^24^ moreover, its occurrence rate is consistent with the literature (i.e., <10%). ^25^ In contrast, the B-G- subgroup is the most likely to experience peri-implant buccal soft tissue dehiscence ^26^ and holds the highest aesthetic risk among all four subclassifications. ^27 , 28^ Therefore, a combination of soft and hard tissue augmentation is necessary for the B-G- subgroup to reduce tissue retraction ^28 , 29^ and buccal bone loss. Moreover, the large ratio of these counterintuitive subgroups (47.6% for the B-G+ subgroup and 23.9% for the B+G- subgroup) may have led to the inconclusive correlation between the buccal bone and gingiva. The prevalence of the B-G+ group suggests that dentists should eliminate the interference of a thick gingiva to detect the genius underlying buccal bone morphology. ^12^ Furthermore, the buccal bone of the B-G+ subgroup was much thinner than that of the B-G- subgroup, leading to a greater risk of postsurgical bone resorption. Therefore, it is recommended that the bone augmentation method be applied in the B-G+ subgroup. ^30 , 31^ From the perspective of periodontal disease, attachment gain after systematic periodontal therapy is more prominent and frequent in thick than in thin gingival biotypes, indicating that this subgroup reacts positively to periodontal therapy. ^32^ Regarding the vulnerability of the B+G- subgroup, soft tissue management techniques, including connective tissue grafts, is advised to be applied to applied to maintain the mid-facial mucosal level. ^33^

The current study assessed only the baseline characteristics of periodontally healthy individuals. Using a digital workflow, an integrated methodology could be adopted to fully quantify the dimensions of the alveolar bone and gingiva in periodontally compromised patients; this would provide more insight into the dynamic correlation between the two tissues and a better understanding of the occurrence, development, and prognosis of periodontal diseases. It is also expected that the detailed subclassification based on the two key parameters of the osseous-gingival ensemble could be applied in clinical practice to provide individualized instructions and a basis for ideal treatment decisions.

## Conclusions

In this study, a population-based canonical correlation analysis revealed a strong correlation between the thickness of the buccal bone and the gingiva of the anterior maxilla. Furthermore, the thickness of the buccal bone and gingiva at 2 mm apical to the CEJ may serve as a scientific-based measurement reference for the presurgical evaluation of immediate implants.
